# Quantifying and Analyzing the Network Basis of Genetic Complexity

**DOI:** 10.1371/journal.pcbi.1002583

**Published:** 2012-07-05

**Authors:** Ethan G. Thompson, Timothy Galitski

**Affiliations:** 1 Institute for Systems Biology, Seattle, Washington, United States of America; 2 Seattle Biomedical Research Institute, Seattle, Washington, United States of America; 3 EMD Millipore Corporation, Billerica, Massachusetts, United States of America; Harvard Medical School, United States of America

## Abstract

Genotype-to-phenotype maps exhibit complexity. This genetic complexity is mentioned frequently in the literature, but a consistent and quantitative definition is lacking. Here, we derive such a definition and investigate its consequences for model genetic systems. The definition equates genetic complexity with a surplus of genotypic diversity over phenotypic diversity. Applying this definition to ensembles of Boolean network models, we found that the in-degree distribution and the number of periodic attractors produced determine the relative complexity of different topology classes. We found evidence that networks that are difficult to control, or that exhibit a hierarchical structure, are genetically complex. We analyzed the complexity of the cell cycle network of *Sacchoromyces cerevisiae* and pinpointed genes and interactions that are most important for its high genetic complexity. The rigorous definition of genetic complexity is a tool for unraveling the structure and properties of genotype-to-phenotype maps by enabling the quantitative comparison of the relative complexities of different genetic systems. The definition also allows the identification of specific network elements and subnetworks that have the greatest effects on genetic complexity. Moreover, it suggests ways to engineer biological systems with desired genetic properties.

## Author Summary

‘Genetic complexity’ is an often-discussed property of genotype-to-phenotype maps, but the term is used vaguely and inconsistently in the literature. We derived a definition of genetic complexity that assigns to every genotype-to-phenotype map a unique quantitative measure of its genetic complexity. This definition allows the meaningful comparison of complexity across systems, and also allows the identification of genetic-network features that contribute most significantly to genetic complexity. We applied this definition to ensembles of Boolean networks. Because all relevant quantities are precisely defined, Boolean networks provide an ideal arena in which to study genetic complexity systematically. Using this approach, we identified relationships between topological properties of networks and their genetic complexity. We also identified features specific to the cell-cycle network of yeast that impart its genetic complexity.

## Introduction

Biologists currently enjoy unprecedented access to genotype and phenotype data, and as the price of DNA sequencing continues to fall and high-throughput automated experimental techniques continue to develop, the amount of data will increase exponentially. The challenge that arises is to extract as much useful information from these data as possible. Molecular, cellular, and behavioral phenotypes are amenable to experimental measurement, but connections to relevant features of the genotype are often out of reach. Likewise, DNA sequencing provides quick and inexpensive access to vast amounts of genotype data, but using the genetic sequence of an organism to predict its phenotype remains a broadly unfulfilled goal. The genotype-to-phenotype map (GPM) encodes the relationship between genetic variations and phenotypes of interest. It is a mapping which assigns to genotypes their corresponding phenotypes. An understanding of GPMs is desirable both to facilitate the prediction of the phenotypic results of genetic perturbations and to identify underlying genotypic features on the basis of phenotypic measurements. The elucidation of GPMs is therefore of primary interest to contemporary genetics.

The properties and construction of GPMs have been the subject of recent studies (see [1]–[3] for review). Another property of GPMs often invoked in the literature is that of ‘genetic complexity’. However, this term lacks a clear and consistent meaning and has variously been taken to mean genetic trait influence that is non-Mendelian, multigenic, additive or epistatic (in the general sense of any genetic interaction). These uses clearly differ from one another and can be contradictory. Furthermore, the lack of a quantitative definition prevents the meaningful comparison of the complexities of multiple genetic systems. The ability to compare the genetic complexity of multiple systems enables the identification of those features and mechanisms that give rise to complexity. Once the relevant features have been identified, the complexity of systems can be controlled. On the genotype side, systems can be engineered to produce greater phenotype resolution and less complexity. On the phenotype side, experimental design can be optimized to maximize the amount of information gained via measurement. A thorough understanding of the genetic complexity of genetic systems is necessary for an understanding of such systems on the whole, and the first step towards this goal is the precise definition of complexity. Here, we derive and investigate a rigorous quantitative definition of the genetic complexity of GPMs.

## Results

**Figure 1:**
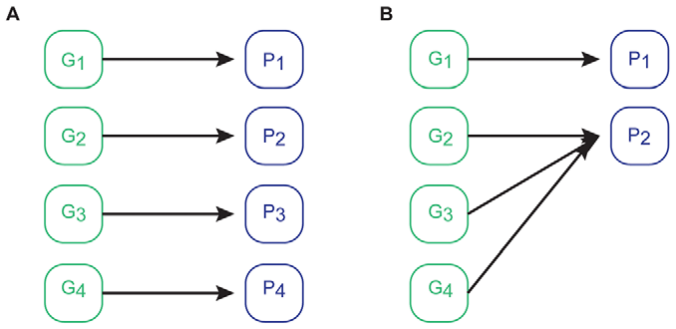
Genotype-to-phenotype maps. (A) An injective mapping illustrating one-to-one mapping of genotype to phenotype. (B) A non-injective mapping illustrating greater genetic complexity.

Consider the two GPMs depicted in Figure 1. The key feature differentiating the two mappings is that the first is injective (one-to-one), whereas the second is not. The non-injective mapping is associated with a greater complexity. This association is in agreement with an information theoretic analysis. The injective mapping has maximal entropy, indicating that much information is gained about the mapping by performing a single measurement. On the other hand, the non-injective mapping has lower entropy, indicating that less information is gained by making a measurement. It is this lack of information gained via measurement that gives biologists the intellectual sensation of “complexity”. The lower entropy of the non-injective map also reflects the fact that such a map has greater order. Complex genetic interactions must be present in the system to give rise to this order. On the other hand, an injective map is less ordered, and a genetic system wherein the genes are acting independently of one another will typically lead to such a map. This provides further justification for the association of non-injectivity with complexity.

**Figure 2:**
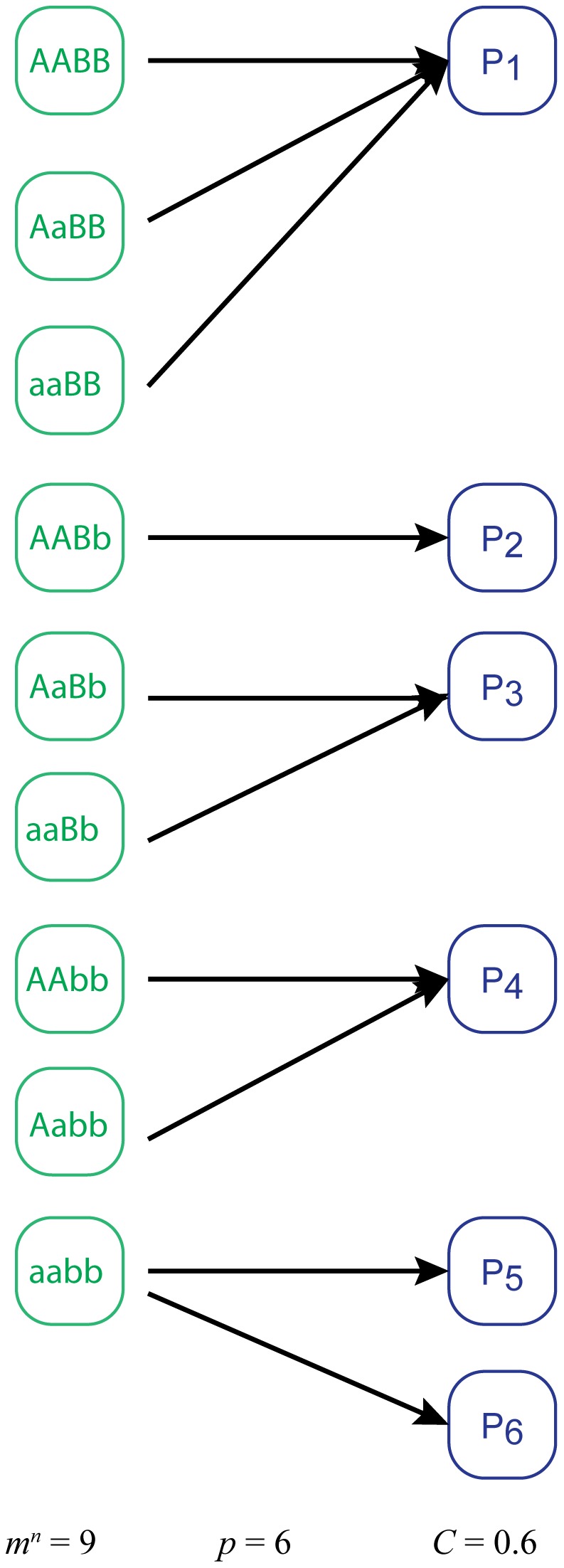
An example genotype-to-phenotype map and the calculation of its genetic complexity. The mapping describes a diploid organism with a phenotype that has two relevant loci (‘A’ and ‘B’), each with two alleles (differentiated by uppercase and lowercase letters).

With this as a guiding principle, a rigorous set-theoretic definition for the genetic complexity of a GPM is derived in Text S1. Because a GPM involves a mapping between two sets, set theory is the appropriate framework with which to formulate a definition of the genetic complexity. The derivation begins with the assumptions of 1) an organism with sexual reproduction and a set of founder genotypes, leading to a set of recombinants, 2) a GPM in which each genotype maps to one phenotype, and 3) the association of complexity with non-injectivity of the GPM, as motivated above. The application of the definition to situations outside this set of assumptions is discussed at length in Text S1. The association of complexity with non-injectivity leads to a definition which is proportional to the difference between a metric of ‘genotype complexity’ and a metric of ‘phenotype complexity’ in the GPM. The bulk of the derivation concerns rigorously establishing the sets of relevant genotypes and phenotypes for a given GPM. The end result is
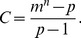
(1)The quantity 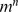 is the genotype complexity of the map: *n* is the number of phenotypically relevant loci in the system and *m* is the geometric mean of the number of phenotypically unique alleles per informative locus. *p* is the phenotype complexity and is equal to the number of measurably distinguishable phenotypes produced by the system across all relevant genotypes. See Text S1 for rigorous formulations of these informal definitions. In practice, the genetic complexity is computed by enumerating the phenotypically relevant genotypes, subtracting the number of phenotypes produced, and dividing by the normalization factor. We thus see that the complexity measures the surplus of genotypic diversity over phenotypic diversity. Our definition therefore captures the increasing complexity of non-injective mappings. The denominator of Equation 1 simply provides a normalization factor that insures that the complexity doesn't grow linearly with the size of the genetic system. In Text S1, we discuss considerations and approaches for the experimental application of Equation 1. An example GPM involving a simple diploid system and its corresponding genetic complexity is shown in Figure 2.

We stress that the genetic complexity is a property a GPM, not of a single genotype and its corresponding genetic network. Thus, the genetic complexity of a GPM is specific to a defined set of genotypes and the experimental protocol and precise parameters of the phenotypic measurements to be carried out on the genotypes. Multiple phenotype measurements can be accommodated readily. In order for the complexity to be a meaningful quantity, the set of genotypes in the GPM should consist of all possible combinations of the alleles under consideration. We refer to this theoretical set of genotypes as a ‘Mendelian library’.

The definition also allows for stochastic behavior. If the phenotype of a particular genotype is measured several times and it is found that two or more distinct phenotypes are produced, then each distinct phenotype will contribute to the quantity *p*. See Text S1 for details. Note that as a consequence the complexity *C* can be negative, indicating that more phenotypes are produced than the number of genotypes in a mapping. GPMs with negative complexity are simply GPMs that are less complex than a GPM with zero complexity. If a single genotype gives rise to multiple phenotypes in a GPM with negative complexity, then not only will the underlying genotype be determinable from a phenotype measurement, but additional information will be gained in the form of which of the multiple phenotypes was realized in a particular case. Although in its initial formulation the definition treats a GPM as a weightless bipartite graph, the definition readily accommodates extensions in which multiple phenotypes arising from a single genotype are weighted, i.e., occur with different frequencies. Phenotypic ambiguity could result also from outlier measurements and from biological (true) noise. Methods for precluding the impact of outliers while accommodating biological noise are presented in Text S1.

In [4], Kruglyak discussed complexity in the context of genome-wide association studies and noted that genetic complexity can be thought of as fractal, with complexity manifest at more than one level. Equation 1 captures much of this idea, with both gene variation and gene combinatorics contributing positively to complexity. However, by the implications of Equation 1, gene variation and gene combinatorics are not complexity per se. Genetic complexity is a surplus of genotype diversity for a given level of phenotype diversity.

Having considered some general features of the definition of *C*, we next wish to investigate its consequences when applied to genetic systems. Though *C* can be estimated from real-world data (Text S1), exact calculation of *C* requires a complete tabulation of a GPM for a system in which the variables in the definition are well-defined. Therefore, as an initial investigation, we applied the definition to model genetic systems for which the relevant quantities can be computed easily. We first carried out a systematic examination of small Boolean network models. We then turned our attention to various features of the complexity of the cell-cycle network of yeast.

### Genetic Complexity of Boolean Networks

Dynamic Boolean network models (“Boolean networks”, henceforth) have long been used to model biological networks [5]–[7]. Boolean networks consist of a set of nodes which, at any moment of time, can either be ‘on’ or ‘off’. For example, the formalism is often applied to genetic networks, where the two states correspond to the gene being expressed or not expressed. Although this is a great simplification of the actual biological state of a genetic network and much dynamical information is sacrificed in this formalism, Boolean networks have proven to be a surprisingly fruitful class of models for capturing the large-scale properties and behaviors of genetic networks (see [8] for a review and further references). Boolean networks are an ideal arena in which to apply the definition of genetic complexity, as they provide models of genetic systems wherein all relevant quantities are well-defined and discrete. In the context of Boolean network models, a genotype is specified by selecting an update function for each node, which determines how the node updates as a function of the network state. Once a genotype is specified, any initial state will flow into an attractor state, which corresponds to a phenotype. The attractor can either be a single state which updates to itself (a fixed attractor) or a series of states through which the network continuously cycles (a periodic attractor). The Boolean network framework allows also for asynchronous update rules for different nodes. In this work, we deal only with synchronous Boolean networks. Details on our employment of Boolean networks are given in the *Methods* section.

As with any mapping, a GPM is not fully specified until the domain on which the mapping acts is delineated. In the case of a GPM, this consists of a specification of the genotypes present in the system. In the abstract arena of Boolean networks, we could conceivably choose any arbitrary set of genotypes to form genotype libraries. However, to identify the effects of some property of the networks on the complexity, we should compare genotype libraries (in which each genotype takes the form of a truth table specifying a Boolean network) that differ only in the property of interest, and that otherwise include all relevant genotypes. In this section, we compare the complexities of network libraries as a function of *order* (number of nodes in the networks), *size* (number of edges in the networks) and *topology*. In all cases, the library of truth tables is generated by selecting the subset of all possible truth tables with the desired number of edges and topology from the set of all truth tables with a given number of nodes. The determination of the edge structure of a network from its truth table is described in the *Methods* section.

#### Complexity by order and size

Initial questions to ask are how the complexity depends on the order and on the size of networks. The natural expectation is that if all other properties of networks are held fixed while the number of nodes and edges is increased the genetic complexity will increase. We obtained results in accord with this expectation.

To test the dependence of complexity on network order, for each order we computed the complexity of the library of networks that comprises all possible genotypes with the appropriate number of nodes. Calculating the complexity of such libraries is computationally tractable only for orders of three or less (for example, there are 10^18^ possible genotypes with order four). We therefore calculated the complexity for all libraries with order less than four. The results are presented in Table S1. The complexity increases monotonically with the order of the library.

Another question to ask is how the complexity of a map depends on the size of the networks considered. To address this, for a given order and size we computed the complexity of the library of networks that comprises all possible genotypes with the appropriate number of nodes and edges. Our method of counting edges in a Boolean network is described in the *Methods* section. The complexities by size for all order-three libraries are given in Table S2. We found that the complexity increases monotonically as the size increases.

#### Complexity by topology

The topology of a genetic network encodes much information about the function of the network. Conserved topological motifs impart similar properties in different biological systems [9], [10]. The importance of network topology to function suggests that there may be a relationship between topology and genetic complexity. The final question that we addressed using small Boolean networks is how complexity depends on the topology of the genotype libraries. To this end, we computed the complexity for libraries that comprise all genotypes within a topology class. A given topology class consists of all genotypes with the same order and the same set of directed edges between nodes. The precise formulation is given in the *Methods* section.

We found that, for a fixed order and size, the relative complexity of topology classes is determined by two pieces of information:

1. The variance of the distribution of in-degrees in the topology class
2. The number of periodic attractors produced by the topology class

The in-degree distribution is the set of numbers of incoming edges incident to each node (see the *Methods* section for further details). Topology classes that have a low-variance distribution of in-degrees (recall that we are comparing classes with equal order and size, and thus the same number of inputs to distribute among the nodes) always have a lower complexity than classes with higher-variance in-degree distributions. This result was found to hold for every combination of network order and size considered, which range from 2 nodes and 1 edge up to 7 nodes and 4 edges. In total, the complexity was computed for 2585 topology classes containing 10^9^ networks. No exception to the above rule was found. This strongly suggests that the rule will continue to hold for yet larger networks. A sample of data supporting this result is given in Table 1.

**Table 1:** The relationship between in-degree distribution and complexity for all topology classes with three nodes and three edges.

Topology Class	In-degrees	C
111 000 000	(3, 0, 0)	78.1818
011 001 000	(2, 1, 0)	4.57143
110 001 000	(2, 1, 0)	2.54545
110 000 100	(2, 0, 1)	2.54545
110 000 010	(2, 0, 1)	2.54545
100 101 000	(1, 2, 0)	2.54545
011 100 000	(2, 1, 0)	1.6
110 100 000	(2, 1, 0)	1.05263
110 000 001	(2, 0, 1)	1.05263
110 010 000	(2, 1, 0)	0.695652
100 100 100	(1, 1, 1)	0
100 100 010	(1, 1, 1)	0
010 100 100	(1, 1, 1)	−0.36364
100 100 001	(1, 1, 1)	−0.46154
010 001 100	(1, 1, 1)	−0.5625
100 001 010	(1, 1, 1)	−0.5625
100 010 001	(1, 1, 1)	−0.65

Topology classes with a higher variance in-degree distribution have greater complexity. The same result holds for all orders and sizes considered. Topology classes are described by three groups of numbers. A ‘1’ in digit b of group a indicates that the node a has an incoming edge from node b.

The difference in complexity associated with different in-degree distributions is most pronounced when one topology class has a node with zero inputs and the second does not. This difference can be understood heuristically as follows. If a topology class has a node with zero in-degree, then that node's update rule does not depend on the current state of any node. The node is immediately locked into whatever state it is in at time *t* = 0. Having one node locked into an invariant state reduces the total number of phenotypes that can be realized by the topology class, resulting in a genotypic surplus. This increases the complexity. Similar reasoning applies to other cases of differing in-degree distributions.

This effect is also active across libraries of different sizes, and accounts for the unexpectedly small difference in Table S2 between the library of size 6 and that of size 7. For an order-three network, if there are 7 or more edges it is not possible to distribute the edges such that any node has zero inputs. Therefore no nodes will be locked into an initial state. Thus, the increase in complexity when adding the seventh edge is smaller than the general trend leads one to expect.

If two classes have the same distribution of in-degrees but a different topology, then the relative complexity is determined by which class realizes more periodic attractors. The class with more periodic attractors has a lower complexity. An example of this is given in Table 2. The relationship between complexity and number of periodic attractors is proven formally in the *Methods* section. It rests on two facts:

1. Any two Boolean-network topology classes with the same in-degree distribution have the same number of genotypes.
2. Every topology class will realize all fixed point attractors.

**Table 2:** The relationship between complexity and the number of loops for all topology classes with in-degrees (1, 1, 1).

C	No. periodic attractors	No. edges in loops	No. loops
0	0	1	1
0	0	1	1
−0.36	4	2	1
−0.46	6	2	2
−0.56	9	3	1
−0.56	9	3	2
−0.65	13	3	3

Topology classes with fewer edges in loops are more complex. If two classes have the same number of edges in loops, the class with fewer loops is more complex. A pictorial representation of these topology classes, with the same ordering, is given in Figure 2.

Because of fact 1 above, the relative genetic complexity of two topology classes with the same in-degree distribution is determined solely by the number of attractors each class realizes. Because of fact 2, the difference in the number of attractors between two such classes is completely determined by the difference in the number of periodic attractors. The topology class with more periodic attractors will have more phenotypes overall, and thus will have a lower complexity.

**Figure 3:**
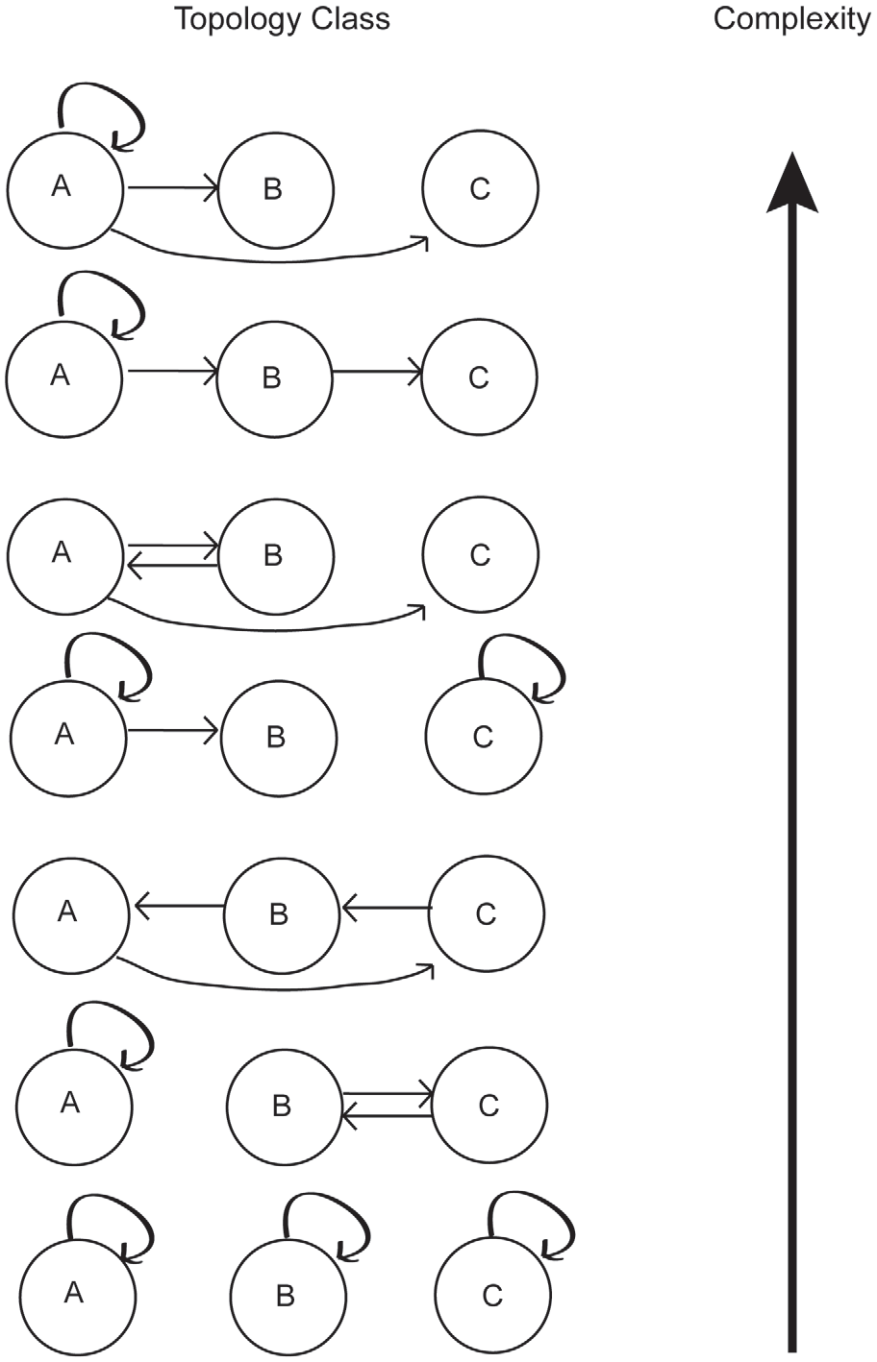
All topology classes with in-degrees (1,1,1), listed in order of decreasing complexity. Topology classes with more loop structures have less complexity. This behavior is summarized in Table 2.

The number of periodic attractors realized provides the second level of structure for the relative genetic complexity of topology classes. However, calculating the number of periodic attractors produced is typically just as difficult as calculating the complexity in the first place. One would like to have a way to predict the relative complexity of two libraries based solely on topological features that can be immediately assessed when presented with two libraries. We found that the number of loops in a topology class provides a qualitative predictor for the number of periodic attractors produced, and thus of the relative complexity of topology classes. An example of the relationship between number of loops and complexity is shown in Figure 3 and Table 2.

It was proposed by R. Thomas that negative feedback loops are necessary for the dynamical realization of periodic attractors [11]. Furthermore, it was recently shown that certain types of loop structures are sufficient to cause dynamical behaviors such as periodic attractors [12]. The relationship between number of loops and periodic attractors identified in this work is in line with these results. The relationship can also be understood heuristically, as a topology class with many loops has many feedback mechanisms. Feedback mechanisms are necessary to create the oscillatory behavior that gives rise to periodic attractors. Though the relationship between number of loops and number of periodic attractors produced does not hold strictly in every case, it does apply in most cases.

Thus, given two topology classes with the same order and the same size, we can make an informed prediction about their relative complexity. If the in-degree distributions of the two topology classes differ, then we know with certainty which will have lower complexity. If the in-degree distributions are the same, the topology class with more loops is likely to have lower complexity. One class of biological networks that exhibit a paucity of loops are gene-regulation networks employing master regulators [13], [14]. These networks tend to utilize a hierarchical structure, whereby information flows strictly from one level (the master regulators) to the next (the regulated target genes). A target of one master regulator can also be a master regulator in its own right, leading to a hierarchical structure with multiple levels. Such networks will feature few loops, and therefore have greater complexity. These hierarchical network structures frequently drive developmental processes that must be robust to environmental fluctuations and that are strongly unidirectional [14]. Our results on small Boolean networks therefore suggest that genetically robust networks in general have greater genetic complexity.

#### Relationship to controllability

In a recent paper [15], Liu *et al.* presented an analysis of the controllability of genetic networks with different topological structures. Though their analysis took place in the framework of continuous linear systems, and can therefore not be rigorously applied to the discrete nonlinear Boolean networks that we have been considering here, their qualitative results suggest a relationship between genetic complexity and controllability. In this context, controllability measures how many nodes of a network must be controlled exogenously in order to be able to realize all possible states in the state space of the network from any possible initial state. A network where fewer nodes must be directly controlled is more controllable.

The authors of [15] found that the minimal number of nodes that need to be controlled in order to fully control the network is equal to the number of ‘unmatched’ nodes. Unmatched nodes are nodes that either have no inputs from other nodes in the network (in-degree of zero), or have inputs only from nodes that have multiple outputs, and thus cannot be individually controlled by any other node in the network. It was shown that the degree distribution plays a primary role in determining the controllability of a network, and that the difficulty of control increases monotonically with degree heterogeneity. These results are highly reminiscent of the relationship between complexity and in-degree distribution that we identified above. We also found that the in-degree distribution plays the primary role in determining genetic complexity, and that an increase in heterogeneity (an increase in in-degree variance) leads to greater complexity. These observations strongly suggest that more genetically complex topologies are also more refractory to exogenous control.

### Complexity and the Cell Cycle Network

**Figure 4:**
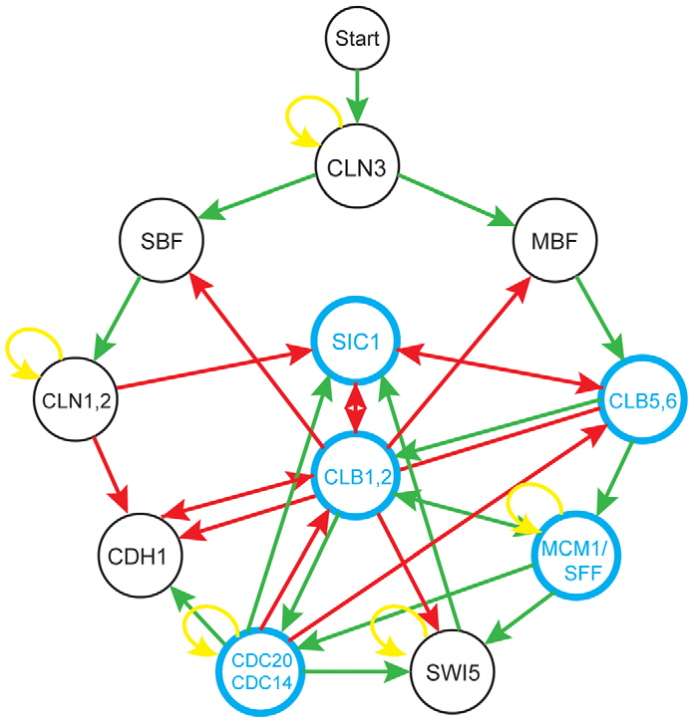
The threshold network formulation of the cell-cycle network of *S. cerevisiae*. The nodes that make the greatest contribution to the complexity of the system are blue.

Having studied the complexity of libraries of generic Boolean networks, we examined the complexity of a specific biological system. The cell cycle network (CCN) of *S. cerevisiae* has been studied extensively as a model network and is well understood. Furthermore, there exists for the CCN a Boolean network formulation [16], allowing the straightforward computation of its genetic complexity. The CCN Boolean network formulation of Li *et al.* [16] uses a threshold network framework. The network is shown in Figure 4 and the details of the threshold network formalism are discussed in the *Methods* section.

Genetic complexity characterizes a library of networks, not a single network, so in order to analyze the complexity of the CCN we must first construct a library which the yeast CCN determines. In the following analysis, for any given threshold network, its corresponding library consists of all threshold networks that have the same topology as the base network and where each node can exist in one of three states: 1) the wild type allele, where the update rule for the node is given by its inputs as defined by Li *et al.*; 2) the null allele, where the node always updates to ‘off’; and 3) a constitutively active allele, where the node always updates to ‘on’. This choice of library is natural, given its biological and experimental relevance, and its symmetry between null and constitutively active states. In this section, when we mention the complexity of a network, we are referring to the complexity of the library generated from the network in the manner described above. For the yeast CCN, which has 11 nodes, this leads to a library of 3∧11 = 177147 genotypes.

We found that for the library generated by the yeast CCN, when all 177147 genotypes were allowed to update from all 2048 initial states, no periodic attractors were produced. Because all fixed attractors were guaranteed to be produced by our construction of the CCN library, the lack of periodic attractors indicates that the complexity of the yeast CCN is maximal. It might seem surprising that a model of a cyclic process gives rise to no periodic attractors. However, this is consistent within the framework of the model, where the G1 phase of the cell cycle is a steady state of the system and initiation of the cell cycle corresponds to an external perturbation. This reflects the biological reality that, at any given time, most cells are not actively progressing through the cell cycle. We then systematically perturbed a number of features of the yeast CCN in order to identify which aspects of the network were most crucial to maintaining its maximal complexity.

First, we calculated the complexity of the 29 networks created by removing a single edge from the network. We found two edges which when removed resulted in a network with lower complexity than the CCN. One of these edges is a positive edge from Clb1,2 to Cdc20, and the other is a negative edge from Clb5,6 to Sic1. Both edges relay information from B-type cyclins, which govern the transition from the G2 phase to the M phase, suggesting that this process contributes significantly to the complexity of the CCN. It is known that the G2/M transition is a key checkpoint of the cell cycle, and that the B-type cyclins play a crucial role in this process [17], [18].

We next systematically perturbed the inputs to each node. For each node, we considered all possible reassignments of its inputs, holding all other features of the network fixed. We then calculated how often the complexity is decreased by perturbing the inputs to each node. We found that there is a clear separation between nodes whose inputs are more or less important to maintaining maximal complexity, as shown in Table 3. The core set of nodes that have the greatest impact on the complexity of the network are highlighted in Figure 4. We found that the nodes whose inputs are more important for complexity are precisely those nodes with out-degree greater than two. This can be understood intuitively, as those nodes with greater out-degree play a larger role in determining the states of other nodes in the network. Perturbing their inputs will therefore have a larger effect on the dynamics of the network as a whole.

**Table 3:** The average effect of perturbations of the incoming interactions for each node in the CCN of *S. cerevisiae*.

Node	Ave. No. of Periodic Attractors per Perturbation	Out-degree
MBF	9	2
SBF	1	2
Cln1,2	3	2
Cdh1	3	2
Swi5	2	1
Cdc20,14	32	5
Clb5,6	40	5
Sic1	24	3
Clb1,2	49	8
Mcm1/SFF	55	3

Those nodes with out-degree greater than 2 make the greatest contribution to the complexity of the system.

We saw evidence in the previous section that networks with more loops tend to give rise to more periodic attractors. Investigations of the CCN also support this conclusion. This can be seen in two ways. In one analysis, we have added all possible single edges to the network and calculated the complexity. When adding an edge, we can also compute how many loops are created, and of what size. We found that those edge additions which lead to the creation of several small loops or very many large loops are more likely to produce periodic attractors (and thereby decrease the complexity) than a random edge addition.

We can also make a connection between the large-scale loop structure and information flow in the CCN and the number of periodic attractors produced. The dominant modes of information flow in the CCN are as shown in Figure S1. The flow of information reflects that fact that the majority of interactions present in the CCN relay information in the pattern indicated. 86% of the edges in the network account for information flowing as indicated. Initial input flows from the top of the network, down either side, and up into the center. There are few edges that connect the bottom of the network to the top, thereby completing the loops in the large-scale information flow. We have found that the addition of edges from the bottom nodes to the top nodes is more likely to decrease complexity than a random edge addition, once again confirming that topologies with more loop structure give rise to lower GPM complexity.

## Discussion

We have formulated a rigorous, quantitative definition of the genetic complexity of a GPM. This definition provides a tool to unravel the properties of GPMs by providing a consistent means of comparing the relative complexities of genetic networks and identifying features of networks that lead to greater or lesser complexity. Genetic complexity is a surplus of genotypic diversity for a given level of phenotypic diversity. Conversely, it is a dearth of phenotypic diversity. It is this dearth that results in the intellectual sensation of surprise when a complex phenotype arises. In biomedicine, such surprises are often unwelcome, for example when the complex phenotype is an adverse reaction to a drug or treatment. With an increased understanding of the quantitative basis of genetic complexity, such surprises can be more predictable, detrimental surprises can be avoided, and the likelihood of salubrious surprises can be increased. Potential applications of the rigorous definition of complexity include evaluating different strategies for collecting data and designing experiments, evaluating the usefulness of statistical methods to determine relevant genes in genome-wide association studies (GWAS) or alternatives to GWAS, and investigating how genetic complexity arises evolutionarily.

We found that the genetic complexity of libraries of Boolean networks increases monotonically as a function of size and order, fulfilling a basic expectation of genetic complexity. We also found that the key determinants of the relative complexity of different topologies are the in-degree distribution and the number of periodic attractors produced by the class (which is qualitatively related to the number of loops in the topology). The central role played by the in-degree distribution in determining the genetic complexity also suggests that those topology classes that are more difficult to control are additionally more genetically complex.

We found that the cell-cycle network of yeast has maximal genetic complexity. In [16], it was shown that the CCN demonstrates a substantial robustness to perturbation. This result therefore also suggests a connection between robustness and genetic complexity. A connection between complexity and robustness was also identified when considering networks with hierarchical structure. The precise nature of the relationship between robustness and complexity should be investigated further. By perturbing the CCN, we identified a core group of nodes which are most responsible for the maximal complexity, and found that interactions involving B-type cyclins make a crucial contribution. Additionally, we reinforced the picture that more loops in a topology leads to lower complexity, in agreement with the association of hierarchical and scale-free structures with complexity.

The definition of complexity should continue to produce other such insights, when applied both to other computational models and to experimental results. The definition allows the identification of those features of genetic interaction networks that lead to more or less complexity and thus leads to a greater understanding of the structure of GPMs in general. Such insights can provide guidance to engineer genetic systems of desired complexity and to design experiments optimally so as to maximize the information gained by performing measurements.

## Methods

### Boolean Networks

In the Boolean network framework, the interacting entities (genes, proteins, complexes, etc.) are represented as binary nodes which can be either active (‘1’) or inactive (‘0’). The current state of the network is then completely specified by stating whether each node is 1 or 0. The network evolves dynamically in discrete time steps. The update rule at time *t* for each node depends on the states of all nodes at time *t*,
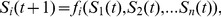
(2)where 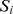
(*t*) is the state of node *i* at time *t*. The function *f* can be represented as a truth table (see Figure S2).

In the context of Boolean representations of genetic interaction networks, specification of a truth table for a node corresponds to specifying how a gene interacts with all other genes. We therefore equate specifying a truth table for a node with specifying an allele for the corresponding gene. In order to fully specify a genotype, then, we must assign a truth table to each node.

Because there is a finite number of states available to a Boolean network of a given size, and because the update rules of the network are fully deterministic, if allowed to evolve in time a Boolean network will necessarily reach an attractor. The attractor can consist of a single state in which the network is forever stuck (a fixed attractor), or it can consist of a series of states that the network continuously visits in the same order (a periodic attractor). For a network with given genotype and initial state, we associate the resulting attractor with a phenotype.

With definitions of genotype and phenotype in hand, we construct a library of Boolean networks according to some unifying principle. We then allow each network in this library to evolve from each possible initial state and record the resulting phenotype. We count the number of unique phenotypes reached and calculate the complexity according to our definition Equation 1. These computations were carried out on a desktop PC, using programs written in C++.

### Topological Features of Boolean Networks

A network can be characterized by its *order* (number of nodes), *size* (number of edges) and *topology* (how those edges are arranged). For a given genotype, the edges of the network can be worked out from the set of truth tables. An edge exists pointing from node j to node i if there exists at least one state such that the update result for i will change if the bit for node j is flipped. Symbolically, an edge from j to i exists if, for some set of 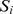,
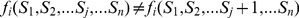
(3)where addition is done modulo 2. Another way to say this is that an edge exists from node j to node i if there is any one case where the update rule of i depends on the state of node j. An example of determining the edge structure from a truth table is given in Figure S2. Our definition of a topology class is then all genotypes (all sets of truth tables) of order n with precisely the same set of edges.

A set of nodes and edges constitutes a topology. Two ways to characterize a topology are by its in-degree distribution and by its out-degree distribution. For an order *n* network, the in-degree distribution is a set of *n* numbers describing how many incoming edges each node has. Likewise, the out-degree distribution is a set of *n* numbers describing how many outgoing edges each node has.

### Formal Proofs

In order to prove that the second level of structure of the genetic complexity of topology classes is determined by the number of periodic attractors produced, we relied on two facts:

1. Two topology classes with the same in-degree distribution have the same genotypic diversity.
2. Every topology class will realize all fixed point attractors.

We prove these two facts now.

First, we show that all topology classes realize all fixed point attractors. Consider any network belonging to a given topology class. This network is fully described by its truth table. For an n-node network, the truth table will have 2^n^ rows and n columns. Each column is the update rule of the nth node, specifying how that node will update for each of the 2^n^ possible states of the network. If we flip the bits of the nth column of the truth table, the nth node will still have inputs from the same set of nodes. Its dependence on these nodes will simply be reversed. Thus, we can flip the bits of any column of the truth table and produce another network in the same topology class. Suppose that we are given any single network representing any topology class. We can construct a member of the same topology class that will realize any state of the network, S = (S_1_, S_2_, … S_n_), as a fixed point attractor, as follows. From the truth table, (S_1_, S_2_, … S_n_) updates to the state (S′_1_, S′_2_, … S′_n_). In order for S to be a fixed point attractor, we must find a truth table in the same topology class such that S_i_ = S′_i_ for all i. Such a truth table can be generated by flipping the bits for each column i in which S_i_≠S′_i_. As shown above, the resulting network will reside in the same topology class, and the state S will be a fixed point attractor of the network. Thus, every topology class will realize all fixed point attractors.

Next, we prove that any two topology classes with the same in-degree distribution have the same genotypic diversity. We consider two topology classes with order *n* and the same in-degree distribution. The number of genotypes in either library is equal to the product of the number of truth tables with the appropriate set of edges for each node. If a given node has in-degree *j*, there is a one-to-one mapping between the sets of all truth tables containing edges from any set of *j* nodes that can be constructed by permuting the node labels. Thus, the number of truth tables accessible to each node depends only on the in-degree of the node and not on the identities of the nodes from which the incoming edges are coming. Therefore, for any two topology classes with the same in-degree distribution, the number of genotypes |*G*| in both classes will be the product of the same *n* numbers.

In order to show that any two topology classes with the same in-degree distribution also have the same genotypic diversity, *m^n^*, we prove that there are no commutative alleles and, therefore, that *m^n^ = |G|*. To prove this, it suffices to show that for any two non-identical Boolean-network alleles, there exists a genetic background for which those two alleles give rise to different phenotypes. Consider two alleles, *A* and *a*, of gene *g*. In the Boolean network framework, this means that there exists at least one state, *s*, for which *A* and *a* have different update rules. Consider state *s* in which the allele *A* updates to 1 and the allele *a* updates to 0. The proof of the converse follows immediately. If gene *g* is 1 in state *s*, then according to the proof of point 2 above, we know there exists a genotype (*A_1_, A_2_,…A,…A_n_*) in the topology class containing allele *A* for which state *s* is a fixed point. But then the genotype (*A_1_, A_2_,…a,…A_n_*), which is also a member of the topology class, does not realize *s* as a fixed point, because gene *g* will update to 0 in this genotype. Therefore, there exists a genetic background (*A_1_, A_2_,…A_n_*) in which *A* and *a* give different phenotypes, and *A* and *a* are not functionally equivalent. The proof for the case where gene *g* is 0 in the state *s* is analogous, with the roles of *A* and *a* switched. Thus, all alleles are functionally unique and *m^n^ = |G|* is the same for all topology classes with the same in-degree distribution.

### Threshold Network Formalism

The CCN as constructed by Li *et al*. utilizes the threshold network formalism. In this formalism, rather than represent update rules by a set of truth tables, which become unwieldy for a large number of nodes, the update rules are specified by a set of arrows. Each arrow points from one node to another, and the arrows must be either positive or negative (represented as green and red arrows, respectively). At each time step, an arrow from node A to node B is active only if node A is on. The update rule for B is determined by looking at all active inputs to B. If more active inputs are positive, then node B turns on. If more active inputs are negative, node B turns off. If there are equal numbers of active positive and negative inputs, then B either remains in its current state, or turns off if it is self-regulating (represented by an arrow pointing from a node to itself). These update rules are summarized mathematically as
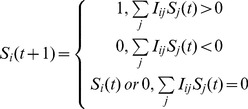
(4)where 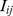 is 1 if there is a positive edge from j to i, −1 if there is a negative edge from j to i, and zero if there is no edge between j and i. For more flexibility, one can also allow the weights 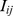 to take on values other than 1 and −1, although we will not consider such cases in this paper.

A network given in the threshold formalism can always be converted to one in the truth table formalism. One potential stumbling block involves a small difference in topological notation between our earlier truth table framework and the threshold network framework. In the threshold network formalism, a self-regulation is represented by an arrow pointing from a node to itself. However, when translated into the truth table formalism, such a node will actually not have an edge pointing from it to itself. Nodes in the threshold network formalism without self-regulation *will* have edges pointing from them to themselves in the truth table formalism, because when their inputs sum to zero they remain in their current state; thus, their update rule depends on their own state.

### Complexity of the CCN

As mentioned in the Results section, for networks in the threshold formalism, we construct libraries of networks by allowing each node one of three possibilities:

1. Update according to the threshold rules given above
2. Update always to off
3. Update always to on

Since there are 11 nodes in the CCN, there are 3∧11 = 177147 genotypes in the CCN library. For each of these genotypes, we cycle through all possible 2048 initial states and find the resulting attractor state. We count the number of unique attractors as the number of phenotypes and calculate the complexity *C*. Once again, the computations are carried out on a desktop PC with programs written in C++. Note that, due to options 2) and 3) above, we are guaranteed to realize all 2048 fixed states, because for each state there exists a network that updates to that state regardless of the current state. For the CCN, only the 2048 fixed attractors are realized, leading to a maximal complexity of 85.54.

For perturbations of the CCN, the calculation is carried out in an analogous manner. We start with the perturbed threshold network, construct the library as above, and count the number of unique phenotypes that result. The 2048 fixed states are always guaranteed to appear and the complexity is fully determined by the number of periodic attractors that are realized.

## Supporting Information

Figure S1 **The general flow of information in the CCN of** ***S. cerevisiae*** **.** Information flows from the upper nodes (colored dark blue) to the lower nodes (colored brown).
(TIF)

Figure S2 **An example truth-table representation of the update rules for a three-node network.** Nodes A, B and C update to A′, B′ and C′ at the subsequent time step. Below is a pictorial depiction of the topology of the particular update rules shown.
(TIF)

Figure S3 **The discretization of continuous phenotype measurements.** Separation along the vertical axis is solely for the purpose of visual clarity. The phenotypes of 8 genotypes, A through H, are plotted on a continuous phenotype measurement axis. Using the method for estimating *p* given in Text S1, the 8 genotypes A–H exhibit *p* = 6 different phenotypes, where B and C share a phenotype, and G and H share a phenotype.
(TIF)

Figure S4 **Two GPMs with equivalent genetic complexity but significantly different structures.** In panel A, each genotype is mapped onto two phenotypes. In panel B, most genotypes are mapped onto a single phenotype, with one exception. The two mappings have equivalent genetic complexity.
(TIF)

Table S1 **The complexity of Boolean networks as a function of order.**
(XLSX)

Table S2 **The complexity of order-three Boolean networks as a function of size.**
(XLSX)

Text S1 **Supplementary info.** Contains the derivation of the definition of genetic complexity and a discussion of the experimental application of the definition.
(DOC)
