# Correction: Quantifying and Analyzing the Network Basis of Genetic Complexity

**DOI:** 10.1371/annotation/a4b94e99-6f8a-433e-9a13-17a9f206eb68

**Published:** 2012-08-15

**Authors:** Ethan G. Thompson, Timothy Galitski

The affiliation of the corresponding author was incorrect. Timothy Galitski is not affiliated with #2 but with #1, Institute for Systems Biology, Seattle, Washington, United States of America.

