# Supplementary figures and images for: Quantifying and Analyzing the Network Basis of Genetic Complexity
Source: PLoS Comput Biol. 2012 Jul 5;8(7):e1002583. doi: 10.1371/journal.pcbi.1002583 (PMC3390359; doi:10.1371/journal.pcbi.1002583)

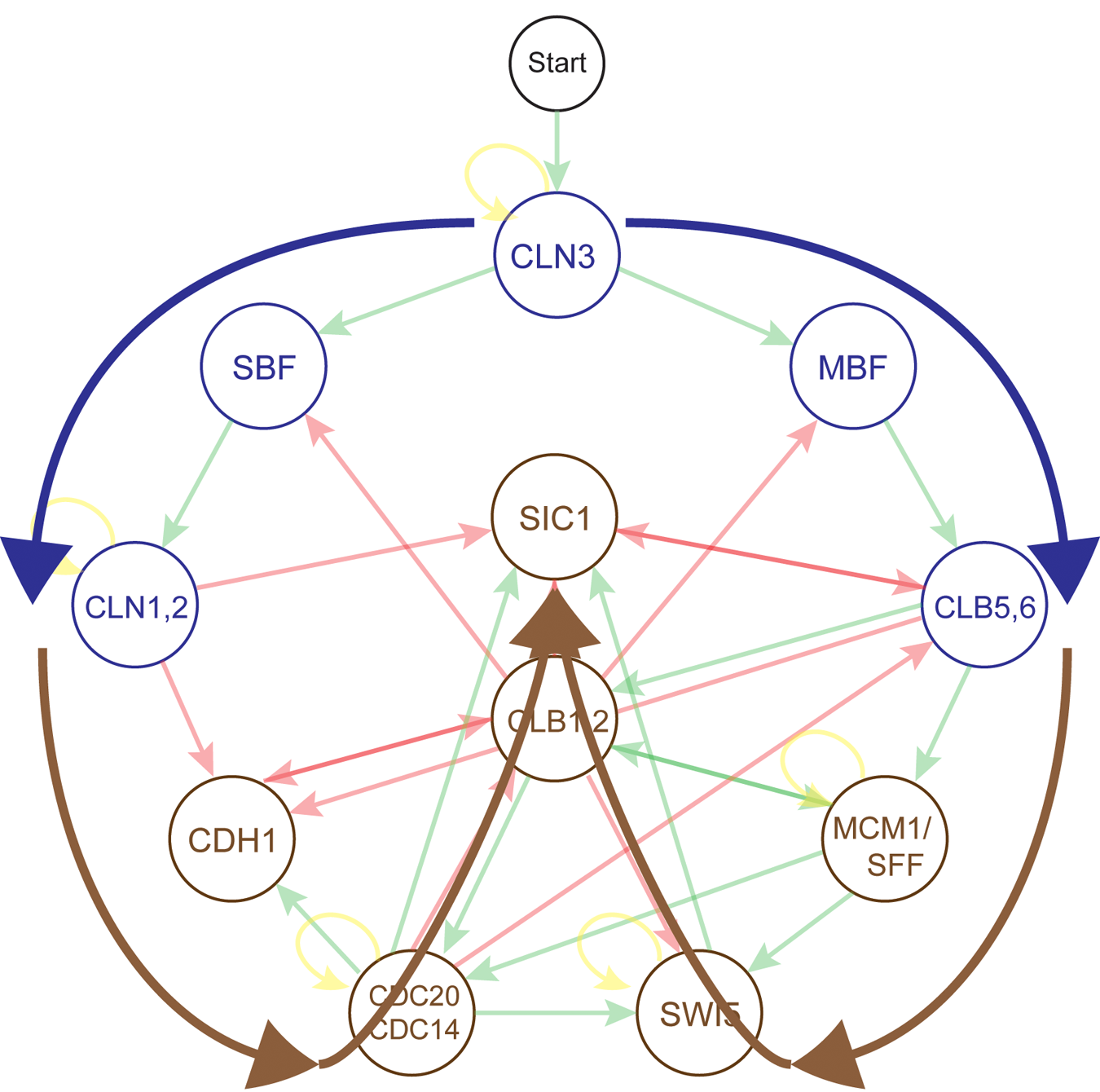

Supplement: Figure S1 — The general flow of information in the CCN of S. cerevisiae . Information flows from the upper nodes (colored dark blue) to the lower nodes (colored brown). (TIF) [file pcbi.1002583.s001.tif]

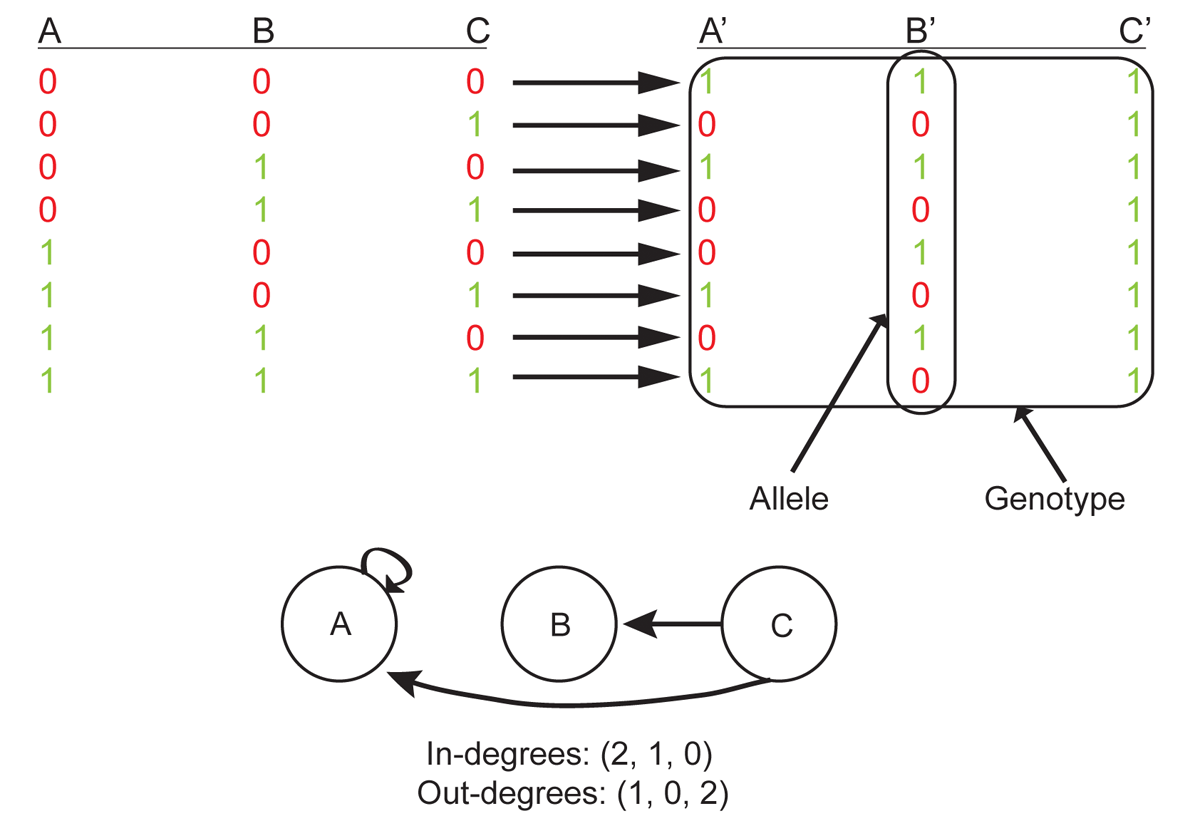

Supplement: Figure S2 — An example truth-table representation of the update rules for a three-node network. Nodes A, B and C update to A′, B′ and C′ at the subsequent time step. Below is a pictorial depiction of the topology of the particular update rules shown. (TIF) [file pcbi.1002583.s002.tif]

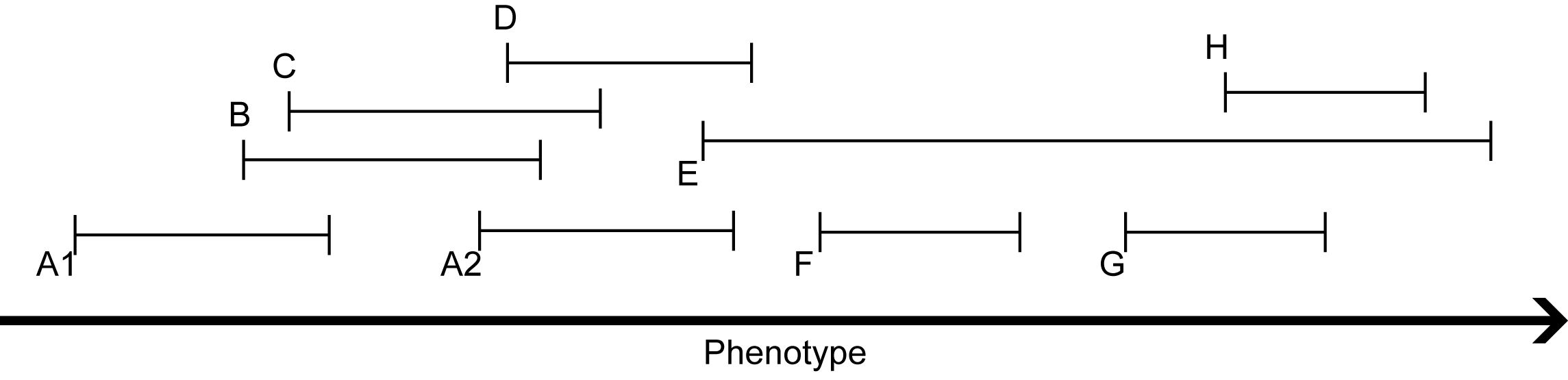

Supplement: Figure S3 — The discretization of continuous phenotype measurements. Separation along the vertical axis is solely for the purpose of visual clarity. The phenotypes of 8 genotypes, A through H, are plotted on a continuous phenotype measurement axis. Using the method for estimating p given in Text S1, the 8 genotypes A–H exhibit p = 6 different phenotypes, where B and C share a phenotype, and G and H share a phenotype. (TIF) [file pcbi.1002583.s003.tif]

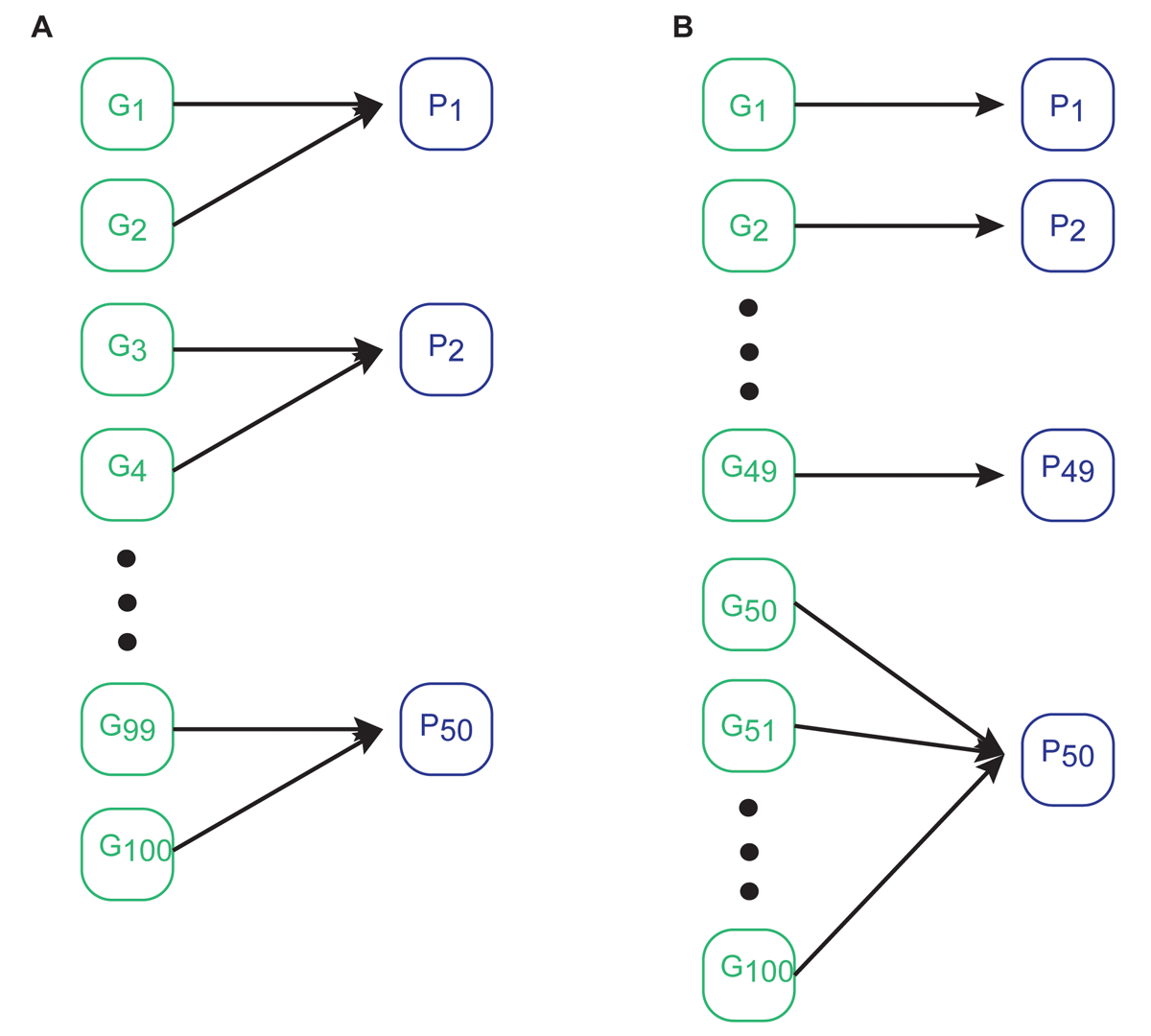

Supplement: Figure S4 — Two GPMs with equivalent genetic complexity but significantly different structures. In panel A, each genotype is mapped onto two phenotypes. In panel B, most genotypes are mapped onto a single phenotype, with one exception. The two mappings have equivalent genetic complexity. (TIF) [file pcbi.1002583.s004.tif]
