# Supplementary material for: Quantifying and Analyzing the Network Basis of Genetic Complexity
Source: PLoS Comput Biol. 2012 Jul 5;8(7):e1002583. doi: 10.1371/journal.pcbi.1002583 (PMC3390359; doi:10.1371/journal.pcbi.1002583)
Supplement: Text S1 — Supplementary info. Contains the derivation of the definition of genetic complexity and a discussion of the experimental application of the definition. (DOC) [file pcbi.1002583.s007.doc]

**Set theoretic derivation of genetic complexity**

Given

1. an organism with sexual reproduction,
2. a set of *fN* founder haploid genomes in *f* founder individuals in a species of ploidy *N*,
3. and the simplifying assumption (this is not required, but is useful to derive the result) of no mutation in the panmictic generation of recombinants derived from the founders,

a genetic locus has a set, *A*, of unique allelic configurations, *A* = {*A1*, *A2*, …}, where each member of *A*, $a\in A$, is an *N*-ploid combination-with-repetition of the founder alleles, and where

|*A*|_max_ = (*fN* + *N* – 1)! / *N*!(*fN* – 1)!, Eq. 1a

which simplifies to

|*A*|_max_ = *f* Eq. 1b

for haploids (*N* = 1) and for homozygous recombinant inbred diploids derived from homozygous founders (e.g., most laboratory mouse strains). Equations 1a and 1b apply likewise to any second locus, for which $b\in B$ = {*B1*, *B2*, …}, and all additional genetically varying loci. For convenience, henceforth we will refer to *N*-ploid allelic configurations as “alleles” and assume haploidy.

Set *G* is the set of all recombinant genotypes that can be derived in principle from the *f* founders. Each genotype is of the form *g* = {*a*, *b*, …}, where $g\in G$, $a\in A$, and $b\in B$. Thus *G* is the Cartesian product of all allele sets

*G* = *A* × *B* × …. Eq. 2

Φ, for which φ$\in$Φ, is the set of all phenotype observations for a single specific trait varying among all genotypes *g*. Φ depends on a specific *G* and a specific phenotype assay. Φ contains discrete (or discretized) phenotype observations. As a simplifying assumption, here we consider genotype-to-phenotype mappings in which each genotype is mapped to a single phenotype. The cardinality of Φ, |Φ|, is the number of phenotypes exhibited by the members of *G*.

Given a specific genotype set *G*, a specific phenotype, and the resulting phenotype-observation set, Φ, there is a set, *M_G_*_Φ_, whose members form the mapping of *G* to Φ. Each member of *M_G_*_Φ_ is of the form *g*→φ. *M_G_*_Φ_ represents the mapping of genotype to phenotype for a specified set of founder genotypes and a specified phenotype assay.

The genotype equivalence class, [g], of $g\in G$ is the set of all genotypes in *G* that are equivalent to *g* given *E*_Φ_, an equivalence relation. *E*_Φ_ specifies that genotypes are equivalent if they map to the same phenotype, φ$\in$Φ. We define *G*_Φ_ ≡ *G*/*E*_Φ_, the quotient set of *G*, where [g]$\in$*G*_Φ_. The mapping of *G*_Φ_ to Φ is bijective, and therefore

|*G*_Φ_| = |Φ| = *p.* Eq. 3

Thus, *p* is a metric of phenotype diversity exhibited by *G*.

The allele equivalence class [a] of $a\in A$ is the set of all members (alleles) of *A* that are equivalent to *a* given *E_M_*, an equivalence relation. *E_M_* specifies that alleles are equivalent if they are commutative in *M_G_*_Φ_. In other words, two alleles of a locus are equivalent if they map to the same phenotypes in all relevant genetic backgrounds. We define *A_M_* ≡ *A*/*E_M_*, the quotient set of *A*, where [a]$\in$*A_M_*. An uninformative locus (in a specific genotype-to-phenotype mapping, *M_G_*_Φ_) is a locus for which [a] = *A* and |*A_M_*| = 1. For informative loci, [a] *A* and |*A_M_*| > 1. *I* is the set of informative loci in *M_G_*_Φ_, and

|*I*| = *n*. Eq. 4

Thus, *n* is the number of informative loci for a specific genotype-to-phenotype map.

The Cartesian product of the quotient sets of all loci is Γ_Φ_, the set of ordered combinations of allele equivalence classes. Thus,

Γ_Φ_ = *A_M_* × *B_M_* × … Eq. 5

In Γ_Φ_ each member is a set of the form {[a], [b], …}. Γ_Φ_ represents *G* without allelic commutativity for a specific phenotype. A simplification of Γ_Φ_ is γ_Φ_, in which only loci in *I* are represented. Note that

|Γ_Φ_| = |γ_Φ_| = *m^n^* Eq. 6

where

$m=(|A_{M}|\times|B_{M}|\times...)^{1/n}$ Eq. 7

is the geometric mean of the cardinalities of the noncommutative quotient sets of alleles of the *n* informative loci (i.e., the geometric mean of the numbers of functionally unique alleles of the relevant genes). The quantity *m^n^* is a metric of genotype diversity that is functionally consequential for a specific phenotype.

If *n* = 1, *M_G_*_Φ_ is a *monogenic* mapping of *G* to Φ. If *n* > 1, *M_G_*_Φ_ is an *n*‑wise *combinatorial* mapping of *G* to Φ.

A *simple combinatorial* mapping is one in which *G*_Φ_ = Γ_Φ_. Thus each genotype equivalence class [g]$\in$ *G*_Φ_ is identical to an ordered combination of allele equivalence classes {[a], [b], …}$\in$ Γ_Φ_, and can be expressed as [g] = {[a], [b], …}. For a simple combinatorial mapping of *G* to Φ, there is a bijective mapping of Γ_Φ_ to Φ, and therefore |Γ_Φ_| = |Φ| = |*G*_Φ_| and *m^n^* = *p*.

A *complex* mapping is a combinatorial mapping that is not simple. For a complex mapping of *G* to Φ, there is a non‑injective yet surjective mapping of Γ_Φ_ to Φ. Whereas in a simple combinatorial mapping *G*_Φ_ = Γ_Φ_, |Γ_Φ_| = |*G*_Φ_| = |Φ| and *m^n^* = *p*, in a complex mapping *G*_Φ_ ≠ Γ_Φ_.  Specifically, |Γ_Φ_| > |*G*_Φ_| = |Φ| and, therefore, *m^n^* > *p*. This observation enables the definition of a measure of the complexity of a genotype-to-phenotype mapping.

The complexity, *C*, of the mapping of *G* to Φ is

$C=\frac{m^{n}-p}{p-1},$ Eq. 8

where *m*, *n*, and *p* have been defined above. The numerator captures the difference between the number of genotypes that can be constructed from noncommutative allele classes and the number of phenotypes. The denominator scales this difference to the supraminimal number of phenotypes. Monogenic mappings (*n* = 1, *m* = *p*) and simple combinatorial mappings (*n* > 1, *m^n^* = *p*) have no complexity (*C* = 0).

**Experimental Application**

Here we consider the application of the definition of genetic complexity to experimental biological systems. The set theoretic derivation of Equation 8 reflects the discrete nature of the elements of a genotype. Thus, Equation 8 defines the complexity of a genotype-to-phenotype map in terms of discrete quantities. This reality necessitates the estimation of *p*, the (discrete) number of distinct phenotypes exhibited by a GPM under consideration. For continuous phenotype measurement data, it is not immediately clear how equality and inequality of measurements should be judged and how the number of unique values of *p* should be tallied. We propose a general solution to this problem.

Suppose that we have carried out an experiment measuring some continuous phenotype and that we have multiple measurements for each genotype. We then have a phenotype distribution for each genotype and can construct a test for equality between two different distributions. For two genotypes g_1_ and g_2_, a simplistic test of equality would be whether the error bars defined by $\mu_{1}\pm\sigma_{1}$and $\mu_{2}\pm\sigma_{2}$ overlap, where
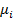
 is the mean phenotype value for genotype *i* and
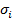
 is its standard deviation. A slightly more rigorous approach would be to assume Gaussian distributions for the repeated measurements of each sample and to apply a t-test. A naïve approach to discretization would then be to apply an equivalence relation based on the test of equality, where all phenotype measurements in the same equivalence class are assigned to the same phenotype class. However, in practice at least some portion of the data is likely to be noisy, and distributions with a large standard deviation will tend to link together many disparate phenotype values, resulting in an underestimate of the number of phenotype classes. We found this was the case in the test data set involving the invasion phenotype of *S. cerevisiae* , where applying the above method resulted in a single phenotype class. This is clearly an inaccurate result.

A solution to this problem is to employ equality in a non-transitive manner. For each genotype, from replicate phenotype measurements one calculates a representative phenotype range that excludes outliers that would otherwise distort the counting of unique phenotypes. An example of such a method is in Drees et al. (2005) [1]. With a sufficient number of replicate measurements, one can use statistical mixture-model methods to detect and define two or more separate phenotype ranges for a single genotype. This accommodates the possibility of a genotype mapping stochastically to more than one phenotype. Figure S3 illustrates the phenotype ranges for 8 genotypes (A through H). There are 9 phenotype ranges because one genotype, A, exhibits two phenotypes, A1 and A2. For each phenotype, one forms a phenotype equivalence class consisting of all phenotypes that are equivalent to the phenotype under consideration via the test of equivalence (e.g. range overlap or t-test). In Figure S3, using the simple range-overlap test, the phenotype equivalence classes are: [A1] = {A1, B, C}; [A2] = {A2, B, C, D, E}; [B] = {A1, A2, B, C, D}; [C] = {A1, A2, B, C, D}; [D] = {A2, B, C, D, E}; [E] = {A2, D, E, F, G, H}; [F] = {E, F}; [G] = {E, G, H}; [H] = {E, G, H}. Then one derives an estimate of the number of unique phenotypes by counting the number of unique phenotype equivalence classes. Those phenotype equivalence classes with identical set membership are counted as a single unique phenotype. In the example at hand, the following phenotype equivalence classes are identical: [A2] and [D]; [B] and [C]; [G] and [H]. See Figure S3 for a graphical illustration of these equivalence class identities. Thus the 9 measured phenotype ranges evince 6 unique phenotypes: A1, A2D, BC, E, F, and GH.

Using Figure S3 for reference, note some key properties of these methods. Due to the high variance of the phenotype measurements for genotype E, using a transitive equality test would lead to an estimate of 1 unique phenotype, whereas the method described above yields an estimate of 6. In addition to the non-transitive approach to phenotype enumeration, the employment of suitable methods, e.g., Drees et al. (2005) [1], to define phenotype ranges avoids the influence of outliers resulting from measurement error while allowing the possibility of wide measurement ranges reflecting true biological noise. Thus, biological noise contributes to the definition and enumeration of phenotypes. This can be seen most clearly in the case of phenotype E. It is counted as a unique phenotype distinct from F, G, and H, all of which are wholly contained within the range of E. Moreover, these methods can accommodate biological stochasticity. A given genotype can exhibit more than one distinct phenotype due to the stochasticity of some underlying biological processes. With suitable methods to detect and define distinct phenotype ranges (e.g., A1 and A2 in Figure S3), these distinct phenotypes contribute to the enumeration of phenotypes and genotype equivalence classes, and consequently to the measurement of the complexity of a genotype-to-phenotype map.

A second major consideration that will arise with experimental application of the definition is its consideration of a complete Mendelian library of recombinant genotypes derived from founder genotypes. Construction and measurement of a complete set of genotypes often will be experimentally challenging. It would be desirable to have methods to estimate the complexity of a GPM based on the data from a partial Mendelian library. To see the difficulty of producing such an estimate, consider the two GPMs shown in Figure S4. The structure of the two maps is clearly different, but they both have the same genetic complexity. Because of the differences in structure of the two maps, partial elucidation of the maps would lead to quite different estimates of the genetic complexity. For example, if a random sample of ten genotypes were measured from both systems, one is most likely to estimate 9 unique phenotypes in the first case, and 6 in the second case. The calculated complexity of the first map would then be 0.125, whereas in the second case it would be 0.8, much closer to the true value of 1.02. However, by performing measurements on a subset of the full library we gain information about the structure of the map that will assist us in computing an accurate estimate of the complexity. In the case of the example, one would be able to conclude that in the first mapping, the distribution of phenotypes is apparently uniform, whereas in the second mapping it is uniform for most of the phenotypes, but one phenotype occurs for approximately half of the genotypes. One could then form a trial distribution based on the structure of the observed part of the map, and fit the total number of unique phenotypes using a maximum-likelihood estimate based on the number of unique phenotypes observed over the measured sample.

1. Drees BL, Thorsson V, Carter GW, Rives AW, Raymond MZ, et al. (2005) Derivation of genetic interaction networks from quantitative phenotype data. Genome Biol 6: R38.
